# Impact of community asymptomatic rapid antigen testing on covid-19 related hospital admissions: synthetic control study

**DOI:** 10.1136/bmj-2022-071374

**Published:** 2022-11-23

**Authors:** Xingna Zhang, Ben Barr, Mark Green, David Hughes, Matthew Ashton, Dimitrios Charalampopoulos, Marta García-Fiñana, Iain Buchan

**Affiliations:** 1 Department of Public Health, Policy and Systems, University of Liverpool, Liverpool, L69 3GB, UK; 2 Department of Geography and Planning, University of Liverpool, Liverpool, UK; 3 Department of Health Data Science, University of Liverpool, Liverpool, UK; 4 Liverpool City Council, Liverpool, UK

## Abstract

**Objective:**

To analyse the impact of voluntary rapid testing for SARS-CoV-2 antigen in Liverpool city on covid-19 related hospital admissions.

**Design:**

Synthetic control analysis comparing hospital admissions for small areas in the intervention population with a group of control areas weighted to be similar for past covid-19 related hospital admission rates and sociodemographic factors.

**Setting:**

Liverpool city, UK, 6 November 2020 to 2 January 2021, under the intervention of Covid-SMART (systematic meaningful asymptomatic repeated testing) voluntary, open access supervised self-testing with lateral flow devices, compared with control areas selected from the rest of England.

**Population:**

General population of Liverpool (n=498 042) and a synthetic control population from the rest of England.

**Main outcome measure:**

Weekly covid-19 related hospital admissions for neighbourhoods in England.

**Results:**

The introduction of community testing was associated with a 43% (95% confidence interval 29% to 57%) reduction (146 (96 to 192) in total) in covid-19 related hospital admissions in Liverpool compared with the synthetic control population (non-adjacent set of neighbourhoods with aggregate trends in covid-19 hospital admissions similar to Liverpool) for the initial period of intensive testing with military assistance in national lockdown from 6 November to 3 December 2020. A 25% (11% to 35%) reduction (239 (104 to 333) in total) was estimated across the overall intervention period (6 November 2020 to 2 January 2021), involving fewer testing centres, before England’s national roll-out of community testing, after adjusting for regional differences in tiers of covid-19 restrictions from 3 December 2020 to 2 January 2021.

**Conclusions:**

The city-wide pilot of community based asymptomatic testing for SARS-CoV-2 was associated with substantially reduced covid-19 related hospital admissions. Large scale asymptomatic rapid testing for SARS-CoV-2 could help reduce transmission and prevent hospital admissions.

## Introduction

Asymptomatic transmission of SARS-CoV-2 has been a major challenge in managing the covid-19 pandemic. Modelling studies based on the original strain had suggested that more than half of transmissions in the community may arise from people without symptoms, whether pre-symptomatic or never symptomatic.^1^ Non-pharmaceutical interventions intended to reduce the risk of transmission from people without symptoms, such as mask wearing, social distancing, and restrictions on travel and access to public spaces and mass gatherings have therefore been necessary. Concerns have, however, been expressed over the potential harms to society and the economy from blunt strategies such as national lockdowns, including the effects of these measures on mental health and health inequalities.^2^

Among other non-pharmaceutical interventions, rapid testing for SARS-CoV-2 antigen using lateral flow devices has now been implemented in many countries for people without symptoms to determine if they are potentially infectious and should self-isolate,^3^ thereby helping to reduce the spread of the virus.^4^ ^5^ ^6^ ^7^ Considerable scientific, public, and political debate has taken place over the mass use of lateral flow devices—the potential harms from false negative and false positive test results, sometimes confusing public health uses to reduce the risk of SARS-CoV-2 transmission with an approximate test of infectiousness with clinical uses to make a diagnosis of covid-19 and the economic opportunity costs.^8^ ^9^ ^10^ Most debates and policies regarding mass testing have, however, lacked controlled comparisons of key outcomes such as hospital admissions for tested versus untested populations experiencing concurrent pandemic phases, with comparable patterns of virus variants and population immunity.

On 6 November 2020, before populations were vaccinated, the UK government piloted the first city-wide voluntary community testing programme, which was open to all residents and workers in Liverpool without symptoms of covid-19.^8^ Box 1 shows the timeline of the pilot. The approach was bold, with an urgent need to generate evidence on how popular such mass testing would be, whether small, controlled environment studies on the accuracy of SARS-CoV-2 antigen lateral flow devices would be reflected in a real world public health setting, and if large scale asymptomatic testing would contain transmission and reduce adverse health outcomes. The early findings from this pilot informed the eventual national roll out of SARS-CoV-2 antigen rapid testing across the UK, as well as internationally.^11^ ^12^

Box 1 **Timeline of Covid-SMART community testing pilot in Liverpool**

**October 2020**

*14 October*
The new three tier system of covid-19 restrictions begins in England, with Liverpool City Region in tier 3, the highest level of restrictions at the time
*31 October*
The UK government offers Liverpool mass testing with military assistance

**November 2020**

*1 November*
Liverpool City Council Covid-19 Strategic Coordination Group with Mersey Resilience Forum accepts in principle but with the freedom to develop a more targeted approach
*2 November*
The military arrives in Liverpool to establish test sites
*5 November*
National lockdown; a communications drive begins in Liverpool on testing, including an interactive map of testing sites and waiting times on Liverpool City Council’s website, and articles put out via digital media in response to testing uptake, feedback on engagement at testing sites, analysis of social media, and commissioned surveys
*6 November*
Six sites open for lateral flow testing (alongside mobile units for polymerase chain reaction (PCR) testing of people with symptoms)
*7 November*
Sixteen sites open for lateral flow testing
*10 November*
First meeting of Department of Health and Social Care’s convened evaluation steering group. Schools-based testing starts
*11 November*
Capacity increased: 37 community sites plus schools, home PCR kits delivered (one-off, unsolicited mailing to sample households), local evaluation group established
*20 November*
Reconfiguration of resources: 15 popular testing sites kept, other resources redeployed to smaller sites in low uptake areas
*23 November*
System for confirmatory PCR changed from national communication and delivery of a home test kit to swabbing at one designated local testing site (with swab kits sent to residents’ homes if they could not travel) and an invitation message tailored to the local area

**December 2020**

*2 December*
Liverpool moved into tier 2, with all surrounding regions in higher tiers or with more restrictions
*3 December*
Handover of management of asymptomatic testing sites from military to Liverpool City Council contractors; targeting becomes more focused as the pilot moves to Liverpool Covid-SMART brand and adapts to fewer covid-19 restrictions
Pilot of visits to care homes in Liverpool begins, and the communications plan shifts priority to test-before-you-go for implementation as the population returned to high transmission risk settings (eg, hairdressers)
Liverpool City Region roll-out of Covid-SMART begins
*4 December*
Test-to-release for some key workers begins
*17 December*
More areas, including Cheshire and Warrington, move into tier 2. Hotels in Liverpool booked heavily with people from London
*31 December*
Move back into tier 3, with all surrounding regions in tier 4

**January 2021**

*4 January*
National lockdown
National roll-out of community testing begins

Although popular overall, uptake of this programme showed substantial differences, with lower uptake among black, Asian, and ethnic minority groups, deprived neighbourhoods, areas at a distance from test sites, and areas containing populations less confident in using internet technologies, such as older people in disadvantaged areas.^9^ Evidence from the pilot showed that lateral flow tests were sufficiently accurate for the intended purpose of community testing, although the number of missed people with high viral load, despite being small, should be taken into account in high consequence settings.^10^ ^13^ The pilot also showed that the expected change in impact of false test results with prevalence should be accommodated in agile, local testing policies.^14^ ^15^ Initial analyses of case rates indicated that community testing in Liverpool was associated with a reduction of around a fifth in infected individuals observed up to the end of December 2020, and that this contrast with other parts of England disappeared as community testing rolled out nationally.^8^ ^10^ Case detection also increased by around a fifth over this period. Causal links between testing and transmission are difficult to make for complex interventions, especially as the pilot was accompanied by a major communication campaign that might have affected risk behaviours of those not testing as well as those using rapid testing services.

In this study, we evaluated whether large scale rapid testing for asymptomatic SARS-CoV-2 infection was effective at reducing covid-19 related hospital admissions.

## Methods

### Setting

Covid-SMART (systematic meaningful asymptomatic repeated testing)^4^ ^12^ was introduced for all people living or working in the city of Liverpool, in the north west of England, from 6 November 2020. Of 151 upper tier local authorities in England, Liverpool is the fourth most deprived,^16^ and at the time, the unvaccinated population had the highest covid-19 case rate in the country. Introduction of Covid-SMART coincided with the start of the second national lockdown (5 November to 2 December 2020).

### Data

Our primary outcome was the weekly number of hospital admissions with a main diagnosis of covid-19 (international classification of diseases 10th revision: ICD-10 code U07.1 for confirmed infections and U07.2 for suspected or probable infections by clinical or epidemiological diagnosis)^17^ in England between 19 November 2020 and 15 January 2021 (intervention period plus two weeks to allow for average lead time from infection to hospital admission), aggregated to middle layer super output areas (MSOA), using Hospital Episode Statistics data provided by NHS Digital covering the period 5 October 2020 to 17 January 2021.^18^

In England, MSOAs are standard geographical units (with an average population of 7200 people) nested within local authorities. We used hospital admissions as the primary outcome, including those in people with a main diagnosis of covid-19 who tested positive for SARS-CoV-2 (ICD-10 code U07.1) or those with a clinical diagnosis of covid-19 (code U07.2).^19^ Hospital admissions as an outcome is less affected by changes in levels of case detection than other outcomes, such as case rates, because the observation probability is less affected by factors such as choice or behaviour, testing capacity, and testing practices. This is important because an objective of the intervention was to increase case detection.

In synthesising controls, we used data on the seven characteristics of local areas that could potentially influence uptake of testing, transmission, effectiveness of control measures, and vulnerability to hospital admission. The selection of characteristics was based on learning from extensive characterisation of differences in covid-19 outcomes and interventions in the study population.^9^ These included the English indices of multiple deprivation 2019—a composite measure of socioeconomic disadvantage,^16^ population density, MSOA population, and the percentage of the population who were aged 70 years or older using mid-year population estimates for 2019 from the Office for National Statistics, the proportion of the population from ethnic minority groups obtained from the 2011 census (the only available data source at this time), and the proportion of the population that had previously been admitted to hospital for a chronic disease (cardiovascular, kidney, or respiratory, or diabetes) between 2014 and 2018 to measure long term prevalence of chronic conditions, using Hospital Episodes Statistics data. To additionally account for potential differences in access to SARS-CoV-2 polymerase chain reaction (PCR) testing between areas before Covid-SMART, we used local authority data available from the UK government covid-19 dashboard on the number of tests per capita in the seven weeks before the introduction of Covid-SMART.^20^ In sensitivity analyses we investigated the inclusion of additional potential confounders in the matching process: proportion of women and the education, skills, and training deprivation domain score from the 2019 index of multiple deprivation.

### Intervention

Covid-SMART was introduced after the UK government selected Liverpool to pilot large scale rapid testing for SARS-CoV-2 antigen in asymptomatic individuals. From 6 November 2020, supervised self-testing with the Innova SARS-CoV-2 rapid antigen lateral flow device was made available to everyone without symptoms living or working in the city of Liverpool. During the initial intensive testing period (6 November to 3 December) the programme was deployed with the assistance of the British Army and was advertised across multiple media channels, with communications also drawing attention to parallel PCR testing for people with symptoms. The initial plan to test 75% of the asymptomatic population in two weeks proved infeasible, but the availability of testing was popular with the public. From 3 December 2020 the service was handed over to Liverpool City Council. The overall aim of the pilot was to reduce or contain transmission of SARS-CoV-2, with testing focusing on the following purposes: test-to-protect vulnerable people and settings (eg, people living in care homes), test-to-release contacts of confirmed infected people sooner from quarantine than the stipulated period (eg, key workers), and test-to-enable careful return to restricted activities to improve public health, social connections, and the economy (eg, mass gatherings). The public was strongly aware of, and had a largely positive attitude towards, Covid-SMART, motivated by shared identity, civic pride, and wanting to protect others.^8^

Individuals who tested positive were instructed to isolate for 10 days according to national guidance and to take a confirmatory PCR test. By 2 January 2021, 33% (n=162 745) of the population had at least one lateral flow test result registered, with 36% (n=57 827) of people testing more than once. Over this period 2113 infected individuals (1.3% of all people tested) were identified using lateral flow tests. Testing was particularly intensive up to 3 December 2020, when 24% (n=117 470) of the self-declared asymptomatic population were tested for the first time in less than a month. The intervention was hypothesised to reduce covid-19 related hospital admissions by preventing onward transmission resulting from the effective isolation of people who tested positive and their contacts, and from the associated publicity raising general awareness of risk behaviours associated with covid-19 and mitigation measures. A proportion of infections prevented as a result of the Covid-SMART intervention would have resulted in admissions to hospital.

### Statistical analysis

We applied the synthetic control method for microdata developed by Robbins et al to estimate the effect of Covid-SMART on covid-19 related hospital admissions.^21^ ^22^ The synthetic control method is a generalisation of difference-in-differences methods, whereby an untreated version of the intervention areas (ie, a synthetic control) is created using a weighted combination of areas that were not exposed to the intervention, and the intervention effect is estimated by comparing the trend in outcomes in the intervention areas with that in the synthetic control areas after the intervention.^23^

As a time lag would be expected between the introduction of Covid-SMART and reduced numbers of hospital admissions, we assumed the minimum plausible period from the start of the testing programme to when an impact on hospital admissions might be expected to be two weeks. We therefore compared the trend in admissions between the intervention and synthetic control areas after 19 November 2020 (ie, 14 days after Covid-SMART started on 6 November). We estimated the intervention effect over two periods: the initial intensive testing period with military support (6 November to 3 December 2020) and the civilian rollout involving fewer testing centres (6 November 2020 to 2 January 2021). The initial testing period coincided with the national lockdown. We used the extended period to understand the extent to which impacts were sustained. From mid-December, asymptomatic community testing was gradually extended to other areas of the country. We therefore limited the follow-up time to 2 January because after that time community testing in Liverpool was no longer being conducted at a higher rate than the rest of England, removing the intervention contrast with control areas (see supplementary figure SF1 for a comparison of lateral flow testing in Liverpool versus the rest of the country).

To construct the synthetic control group, we derived calibration weights to match the 61 MSOAs in Liverpool to areas outside Liverpool before the introduction of Covid-SMART. The weighting algorithm derives weights for all MSOAs outside Liverpool that meet two constraints. Firstly, the weighted average of each of the seven local area characteristics, (MSOA population, index of multiple deprivation, population density, proportion of the population aged 70 years or older, proportion of the population from ethnic minority groups, five year hospital admission rate for chronic conditions, and PCR testing rate before intervention) in the control group is equal to the average for Liverpool.^21^ Secondly, the total number of covid-19 related hospital admissions in the control group equals the number of hospital admissions in Liverpool for each of the seven weeks before 19 November (preintervention period). Matching the synthetic control group on preintervention trends in hospital admissions for covid-19 was important to minimise potential differences in unobserved characteristics. Those MSOAs allocated a non-zero weight by this process then contribute to the synthetic control group.

From the pool of MSOAs used to construct the synthetic control, we excluded MSOAs within the Liverpool City Region (139 MSOAs in five local authorities, other than Liverpool) to avoid spill-over effects of community testing on neighbouring areas. As we wanted to estimate the effect of Covid-SMART alone, we treated Liverpool as the intervention group and excluded from the control group any local authorities with higher than the average weekly lateral flow testing rate of 1 per 100 population between 6 November 2020 and 2 January 2021. This removes influences of any unknown pilots of lateral flow tests among the 15% of non-Liverpool local authorities testing above this level (see supplementary figure SF2). Overall, 142 MSOAs were excluded in this way (2.2% of all non-intervention MSOAs), leaving 6290 MSOAs to comprise the synthetic control group.

The average treatment effect for the treated was then estimated as the difference in cumulative number of hospital admissions in the post-intervention period in Liverpool compared with the (weighted) cumulative number of admissions in the synthetic control group. We used permutation samples, by repeating the analysis through 250 placebo iterations randomly allocating MSOAs outside Liverpool to the intervention group, to estimate the sampling distribution of the treatment effect and calculating permuted P values and 95% confidence intervals.^22^

After the national lockdown ended on 2 December 2020, a three tiered system of local restrictions was implemented. Liverpool entered less stringent tier 2 (high alert) restrictions owing to lower levels of covid-19, while most similar areas entered tier 3 (very high alert) restrictions, which appeared to have a relatively large impact on transmission.^23^ We therefore adjusted our analysis for the extended period (6 November 2020 to 2 January 2021) to remove the effect of the tier 3 restrictions relative to tier 2 restrictions in the synthetic control group. Extending our previous analysis,^23^ we found that tier 3 restrictions were associated with a reduction in hospital admission rates by 17% (95% confidence interval 13% to 21%) relative to tier 2 restrictions, and that these effects started around the 20 December 2020 and extended to the 21 February 2021. We therefore adjusted the cases in tier 3 areas upwards by this percentage during this period before deriving weights to provide a synthetic control group reflecting transmission conditions that were experienced in Liverpool at that time (see supplementary file part 2 for how this adjustment was estimated).

All analyses were performed using R version 4.0.3 and the Microsynth package.^21^

### Sensitivity analyses

In sensitivity analysis we repeated the synthetic control models for the upper and lower plausible estimates of the potential effect of less stringent tier 2 restrictions in Liverpool. These were based on the upper (21%) and lower (13%) bounds of the 95% confidence interval of our estimate of the tiered effect (see supplementary file part 4 for results). We also replicated the analysis without excluding places with mean weekly lateral flow test rates >1 per 100 population (see supplementary file part 5 for results).

To account for the potential bias from our synthetic control group being constructed from a dispersed non-adjacent set of neighbourhoods, while our intervention group was the contiguous neighbourhoods of Liverpool, we conducted a sensitivity analysis. In this analysis we used a different synthetic control approach with aggregated local authority data, comparing Liverpool to a group of similar cities and towns (see supplementary file part 7 for results). We also conducted sensitivity analyses to check whether our choice of matching variables was robust by incorporating the proportion of women (see supplementary file part 8) and the education, skills, and training deprivation domain score rather than the composite score of the index of multiple deprivation (see supplementary file part 9), respectively.

### Patient and public involvement

The implementation of Covid-SMART in Liverpool involved regular focus groups with residents run by Liverpool City Council with the University of Liverpool. Further details are available at https://www.liverpool.ac.uk/coronavirus/research-and-analysis/covid-smart-pilot/.

## Results

Table 1 presents summary statistics for the 61 MSOAs that make up Liverpool and the pool of MSOAs in the rest of England from which the synthetic control group was constructed (see supplementary file part 5, tables SF3 and SF4 for further details). Liverpool has markedly higher levels of deprivation, higher population density, higher proportion of the population previously admitted for a chronic disease, lower proportion of the population aged 70 years or older, and a lower proportion of the population from ethnic minority groups. In the seven weeks before the introduction of Covid-SMART, Liverpool had a higher number of PCR tests per capita and higher covid-19 related hospital admissions and case rates than the average for the rest of England. The weighted average of each matching variable of the seven local area characteristics (MSOA population, deprivation, population density, proportion of the population aged 70 years or older, proportion from ethnic minority groups, five year hospital admission rate for chronic conditions, and PCR testing rate before intervention) achieved an exact match between the intervention (Liverpool) and synthetic control areas. Supplementary table SF4 compares the total number of covid-19 related hospital admissions in the control group with Liverpool for each of the seven weeks before 19 November (preintervention period) and shows a perfect match between the intervention group and synthetic control group. Supplementary figure SF4 shows the geographical pattern of these weights when constructing the synthetic control group. Many MSOAs allocated a non-zero weight are near Liverpool, such as Warrington, Wigan, Wyre, and Manchester, whereas others cluster in the north east (Northumberland, Newcastle upon Tyne, South Tyneside, Sunderland, and Middlesbrough), in Yorkshire and The Humber (Barnsley, Leeds, north east Lincolnshire, Rotherham, and Sheffield), or disparately (Solihull, Nottingham, Hammersmith (London), Fulham (London), Torbay, Southampton); see supplementary figure SF4.

**Table 1:** Comparison between Liverpool and MSOAs in the rest of England used to construct the synthetic control group

Characteristics	Liverpool	MSOAs in rest of England
No of MSOAs	61	6290
Total population	498 042	52 330 147
MSOA population†	8165	8320
Index of multiple deprivation 2019 score†	43	21
Population density (No of people per hectare)†	55	36
Population aged ≥70 years (%)†‡	11	14
Ethnic minority group (%)†§	11	14
Population with ≥1 admission for chronic disease†¶	24	20
No of PCR tests per 100 000 population†**	3572	2552
Average weekly hospital admissions per 100 000 population for covid-19**	26	9
Weekly covid-19 cases per 100 000 population**	464	203

MSOA=middle layer super output areas; PCR=polymerase chain reaction.

The synthetic control group excludes those within Liverpool City Region or with a high rate for lateral flow testing.

† Matched local area characteristics used to construct the synthetic control group.

‡ Calculated using mid-year population estimates for 2019 from Office for National Statistics.

§ Data obtained from the 2011 census.

¶ Based on Hospital Episode Statistics data between 2014 and 2018.

** Data refer to the preintervention period from 5 October to 5 November 2020.

**Fig 1:**
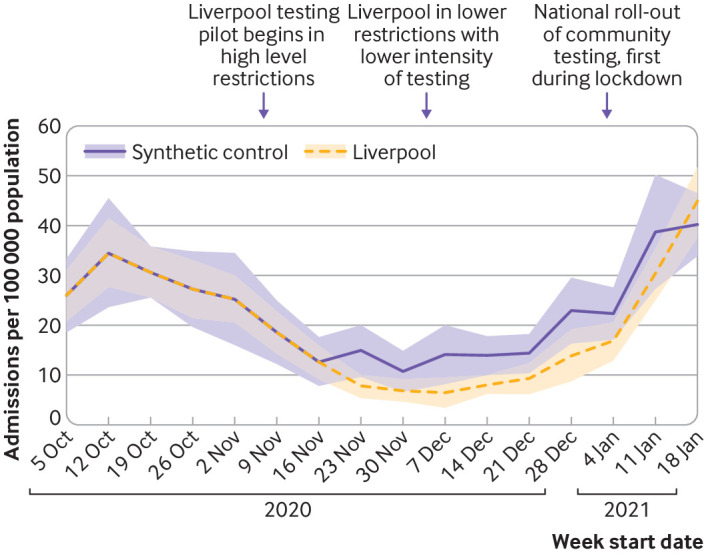
Trend in weekly covid-19 hospital admission rates in middle layer super output areas (MSOAs) in Liverpool city compared with a synthetic control group constructed from the weighted average of MSOAs outside Liverpool City Region without community testing. Community testing pilot for SARS-CoV-2 was introduced in Liverpool on 6 November 2020, followed by tier 2 covid-19 restrictions on 3 December 2020, before the national roll-out of community testing in lockdown on 3 January 2021. Adjustments are for the estimated effects of December 2020s tier 2 versus tier 3 restrictions on covid-19 related hospital admissions

Figure 1 shows the trend for the average covid-19 hospital admission rates from 5 October 2020 until 17 January 2021 across MSOAs in Liverpool, and the synthetic control group. Owing to an exact match in calibration weights, trends were identical between the intervention group and synthetic control group in the preintervention period (5 October to 5 November 2020). Trends began to diverge two weeks after the introduction of Covid-SMART, however, with hospital admissions being lower in Liverpool than in the synthetic control group. The lower trend in Liverpool continued throughout December before rising sharply in January to match that of the synthetic control group, which coincided with the expansion of community testing to other areas.

Table 2 shows the results of the synthetic control analysis, indicating estimated effect of Covid-SMART on covid-19 related hospital admissions. We present the estimated effects for three different models: model 1 assumes no effects of the tier 3 restrictions during the initial intensive testing period with high level restrictions (6 November to 3 December 2020); model 2 also assumes no effects of the tier 3 restrictions but extends the initial period to 2 January 2021; and model 3 covers the same extended period as model 2 (6 November to 2 January 2021) but seeks to adjust the effects of tier 3.

**Table 2:** Estimated effects of Covid-SMART community testing on covid-19 related hospital admissions from synthetic control analysis, under alternative assumptions over the effects of a lower level of restrictions in Liverpool city, December 2020

Model	Intervention period	Assumed reduction in admissions from tier 3 *v* 2 restrictions (%)	Difference in hospital admissions (Liverpool *v* control)		P value
			% (95% CI)	Absolute (95% CI)	
1	6 Nov to 3 Dec 2020	Nil	−43 (−57 to −29)	−146 (−192 to −96)	<0.001
2	6 Nov 2020 to 2 Jan 2021	Nil	−16 (−27 to 0)	−133 (−239 to −3)	0.07
3	6 Nov 2020 to 2 Jan 2021	17 (central estimate)	−25 (−35 to −11)	−239 (−333 to −104)	<0.001

CI=confidence interval.

Over the initial intensive testing period (6 November to 3 December 2020) (model 1), admission rates in Liverpool were 43% lower than in the synthetic control group (95% confidence interval 57% lower to 29% lower). In absolute numbers this 43% reduction is the equivalent of 146 (96 to 192) fewer admissions in the period up to 3 December 2020.

When extending the analysis to the period up to 2 January 2021 (model 2), we observed a smaller, estimated effect of Covid-SMART in reducing admissions by 16% (95% confidence interval 27% lower to 0%), or −133 (−239 to −3) in absolute terms, in Liverpool compared with control areas. After adjusting for the anticipated effect of tier 3 restrictions on covid-19 hospital admissions using our central estimate, the impact of community testing was observed to increase, and hospital admissions reduced by 25% (95% confidence interval 35% lower to 11% lower), or 239 (104 to 333) in absolute terms (model 3).

Sensitivity analyses using the upper and lower plausible estimates of the potential tier effect showed coherent and similar results with those of model 3 (see supplementary file part 4 for more details). We also repeated our analysis by including areas with mean weekly lateral flow testing rates >1 per 100 population; findings were similar to those in table 2 (see supplementary file part 5 for more details). We found similar results in a sensitivity analysis using the aggregated local authority level data (see supplementary file part 7), adding the proportion of women (see supplementary file part 8), and using the education, skills, and training deprivation domain score rather than the composite score of index of multiple deprivation (see supplementary file part 9).

## Discussion

### Principal findings

This study found that the introduction of community testing for SARS-CoV-2 in Liverpool, ahead of its wider implementation across the UK, was associated with a reduction in covid-19 related hospital admissions compared with what would have been expected in the absence of this intervention. This effect was observed to be greater when analysis was restricted to the first month of implementation, when testing was more intensive through military assistance and before Liverpool entered a lower level of restrictions than most other cities, at the same time as the alpha variant of SARS-CoV-2 spread nationally. This suggests that widespread community testing has an effect at reducing transmission and consequently covid-19 related hospital admissions. We also found similar effects when we explored the impact of the early roll-out of community testing across the wider Liverpool City Region using an equivalent synthetic control analysis, where we estimated a 32% (95% confidence interval 22% to 39%) reduction in covid-19 related hospital admissions.^8^ Early findings from this pilot informed the national roll-out of SARS-CoV-2 antigen rapid testing across the UK and have influenced policies internationally.^11^

### Strengths and limitations of this study

Our analysis has several strengths. We were able to use small area data to construct a control group with similar characteristics to our intervention population. The synthetic control approach ensured that control areas were similar for both level and prior trends in hospital admissions, indicating that these areas were likely to have been affected by similar SARS-CoV-2 transmission patterns before the introduction of Covid-SMART in Liverpool. This is important as the parallel trends assumptions of simple difference-in-differences methods are not sufficient for analysis of infectious diseases, where the rate of change is intrinsically linked to the levels of infection at baseline.^24^

Our study also has several limitations. Firstly, although we were able to match areas to ensure a good balance of potential confounding factors before the intervention, it is possible that concurrent changes in the intervention or control populations, or both, could bias the results. The major policy change that affected transmission at this time was the introduction of tiered restrictions, and we have sought to adjust for these in our analysis and present sensitivity analysis assuming different effects of this policy on transmission. The adjustments we made for these differences in restrictions assumed that the effect of tier 2 restrictions on SARS-CoV-2 transmission in Liverpool was the same as the average effect across tier 2 areas in England. The effect could, however, have been greater in Liverpool, because unlike other tier 2 areas, most of the areas surrounding Liverpool were in tier 3. The lower restrictions in Liverpool might have encouraged populations from surrounding areas to use the restaurants and other facilities open in Liverpool that were closed in their own areas at the time. In addition, more restrictive tier 4 restrictions were applied in some other areas within the synthetic group in late December 2020. Therefore our estimates for the effect of Covid-SMART may be overly conservative. Secondly, there are potential spill-over effects, with community testing affecting transmission beyond Liverpool—particularly as testing was available to people working in Liverpool. We sought to account for that by excluding surrounding areas from the control group. Thirdly, our synthetic control group was made up of a non-adjacent set of neighbourhoods, which in aggregate had shown similar trends in hospital admissions to those in the contiguous neighbourhoods of Liverpool—although we found similar results when matching Liverpool to larger local authority areas (see supplementary file part 7). In our analysis we did not consider potential effects on transmission of these differences in the spatial dispersion of the intervention and control neighbourhoods. One might expect that this would lead to a more rapid increase in transmission in Liverpool compared with the synthetic control areas, as neighbourhoods in Liverpool tended to be adjacent to areas with high case rates, whereas the synthetic control neighbourhoods tended to be adjacent to areas with lower case rates—that is, this would be expected to dilute the intervention effect. Fourthly, we were only able to use data on small neighbourhood areas rather than on individuals and therefore were not able to investigate how effects of community testing varied by individual or household characteristics. Fifthly, the causal inference cannot be applied to the use of rapid antigen tests alone because the extensive communication required to implement community testing may have affected covid-19 risk behaviours in those not taking tests.

Finally, our study predated vaccination and the omicron variant with higher transmissibility but lower hospital admissions.^25^ ^26^ Despite the World Health Organization’s recommendation to find, test, treat, and isolate for containing communicable diseases, the UK government ended community testing on 1 April 2022. Before that, people had free and ample access to lateral flow devices and were encouraged to test frequently. Our study also reflects supervised self-swabbing at testing centres, whereas home testing became the norm. Therefore, care should be taken when interpreting the findings of this study in the context of different epidemics, immunity, and testing.

### Policy implications

Debate about the potential benefits and harms of mass testing using lateral flow tests in response to covid-19 has been widespread.^27^ Mass testing is increasingly recognised as an important non-pharmaceutical intervention for identifying infectious people.^4^ ^28^ ^29^ ^30^ Given the importance of asymptomatic transmission,^1^ measures that shorten the time between testing and results have the potential to disrupt transmission through timely isolation of the most infectious people and their close contacts. Criticism of this approach, however, has focused on the accuracy of tests, potential lack of adherence to self-isolation of those identified as infectious and their contacts, and insufficient evaluation before roll-out.^5^ The experience in Liverpool indicates that widespread community testing is feasible and can detect infectious people who would not otherwise have been identified.^8^ Survey results from Liverpool indicated that a high proportion of infectious people who were identified reported that they did self-isolate after testing positive.^8^

It is plausible that the main effect in our analysis is causally related to the Covid-SMART intervention, especially as the study period pre-dates the main roll-out of covid-19 vaccination. Over the full follow-up period a 25% reduction of what would have been without Covid-SMART is the equivalent to an absolute reduction of about 239 hospital admissions in Liverpool. Assuming an infection hospital admission ratio of 3.5%,^31^ a reduction in 239 admissions would suggest that around 6829 infections would need to be prevented to reduce hospital admissions by this amount. In other words, if this effect was causal, the isolation among the 5110 test positive individuals found in the Covid-SMART study prevented around 6829 infections by identifying infectious people sooner and disrupting transmission. The prevented infections may have been directly due to the isolation of those initial 5110 infectious people or from the onward transmission through their contacts observed before 2 January 2021.^32^

### Comparison with other studies

Although many countries have implemented large scale rapid testing for SARS-CoV-2 antigen, evaluation of its impact on transmission and hospital admissions have been limited. A modelling study found that one round of mass testing might reduce daily infections by 20-30% but that these effects are likely to be relatively short term, with infections returning to pre-mass testing levels shortly after an initial wave of testing.^6^ Analysis of population-wide testing in Slovakia indicated that mass testing was associated with a 58% reduction in transmission, although this analysis was not able to distinguish between the impact of mass testing and other control measures that were introduced at the same time.^7^ Our study findings are broadly consistent with these estimates.^14^

Similar to previous studies, we found that effects seem to have been greatest early on in the programme when large numbers of tests were administered to a large number of people within a relatively short period.^6^ ^33^ Others have also highlighted the importance of combining rapid testing in a large proportion of the population along with minimising delays to testing to control transmission, with modelling indicating that this can compensate for lower test sensitivity.^33^ However, the use of asymptomatic testing to control transmission will be undermined if test positive people are unable, or disinclined, to isolate—for example, if there are financial penalties to isolation. Effectiveness will also be reduced when, as we found in Liverpool, uptake of testing tends to be lower in populations where transmission tends to be higher (eg, among more deprived groups).^9^ Our findings, however, suggest that even when uptake is unequal and barriers to effective isolation exist, widespread community testing can potentially reduce transmission and subsequent hospital admissions at least in the short term. Further strategies for asymptomatic testing in the community should aim to maintain high levels of repeat testing, particularly targeted at high risk groups. Combining this with other control measures could allow control of SARS-CoV-2 while maintaining social and economic activity.

### Conclusion

The voluntary, city-wide SARS-CoV-2 rapid antigen testing pilot in Liverpool was associated with a substantial reduction in covid-19 related hospital admissions. Community asymptomatic testing for SARS-CoV-2 antigen with lateral flow devices has been a useful addition to the measures for mitigating risks of covid-19. The success of such control measures relies on high levels of uptake and effective support to enable isolation of infectious people and their close contacts. For successful public health responses to covid-19, large scale community testing is more than a test. It is a complex intervention comprising communication, technology, and social responses, which when combined may reduce SARS-CoV-2 transmission beyond the individual effects of tests on early identification and isolation of test positive individuals.

**What is already known on this topic**

Previous studies on managing the spread of SARS-CoV-2 identified asymptomatic transmission as major challenges for controlling the pandemic
Along with non-pharmaceutical measures, many countries rolled out population based asymptomatic testing programmes to further limit transmission
Evidence on whether large scale voluntary testing of communities for covid-19 reduces severe disease by disrupting transmission is lacking

**What this study adds**

This study found that large scale rapid antigen testing of communities for SARS-CoV-2, within an agile local public health campaign, can potentially reduce transmission and prevent hospital admissions
The policy implications are that testing during a pandemic is best integrated within local public health programmes, supporting those required to isolate and adapting to prevailing biological, behavioural, and environmental circumstances

## Data Availability

The small area covid-19 hospital admissions data were made available by NHS Digital under data sharing agreement DARS-NIC-16656-D9B5T-v3.10 and are available through application to NHS Digital. All other data are publicly accessible and code is available via the Liverpool City Region Civic Data Cooperative GitHub public repository (https://github.com/civicdatacoop/COVID-SMART-in-Liverpool/tree/main ).

## Web extra

Extra material supplied by authors

Supplementary material: additional information parts 1-9, including tables and figures

## Notes

Contributors: XZ and BB are joint first authors. IB led the overall study, provided clinical and public health expertise on interpretation of findings, and drafted, critically reviewed, and finalised the paper. BB conceptualised the analytical plan, analysed the data, interpreted the results, drafted the paper, and critically reviewed updated versions of the paper. BB and IB are guarantors of the study. XZ cleaned and analysed the data and drafted and revised the paper. DC drafted the paper and helped with interpretation of findings. MGF provided expertise on study methodology and critically reviewed the paper. MG and DH contributed to the analyses, helped with interpretation of findings, and critically reviewed the paper. The corresponding author attests that all listed authors meet authorship criteria and that no others meeting the criteria have been omitted.

The study guarantors (BB and IB) affirm that the manuscript is an honest, accurate, and transparent account of the study being reported; that no important aspects of the study have been omitted; and that any discrepancies from the study as originally planned have been explained.

Dissemination to participants and related patient and public communities: The COVID-SMART pilot involved regular focus groups with residents run by Liverpool City Council in collaboration with University of Liverpool as detailed at https://www.liverpool.ac.uk/coronavirus/research-and-analysis/covid-smart-pilot/. The emerging findings of this study have been shared through formal scientific communication platforms, public media and social media, and relevant government agencies in the format of press releases, online engagement, meetings, reports, and a preprint manuscript.

Provenance and peer review: Not commissioned; externally peer reviewed.
