# Supplementary material for: Impact of community asymptomatic rapid antigen testing on covid-19 related hospital admissions: synthetic control study
Source: BMJ. 2022 Nov 23;379:e071374. doi: 10.1136/bmj-2022-071374 (PMC9682337; doi:10.1136/bmj-2022-071374)
Supplement: Supplementary file 1 — Supplementary material: additional information parts 1-9, including tables and figures [file zhax071374.ww.pdf]

Supplement

Part 1: Lateral flow testing rates over time.

Trends in the number of tests over time (Figure SF1) reflect initial high uptake during the initial push, declining following planned withdrawal of military assistance shortly after Liverpool's move into less stringent (Tier 2) local restrictions (announced 26th November 2020, enacted 2nd December 2020). Uptake remained initially low in December, before a sharp increase in the week before Christmas as individuals may have sought tests before mixing among Christmas bubbles. High demand was sustained after Christmas and into the national lockdown (starting 6th January 2021).

Figure SF1. Trend in lateral flow tests per 100 population each week in Liverpool and in the rest of England. The vertical line marks the rollout of the asymptotic community testing pilot in Liverpool on 6th November 2020.

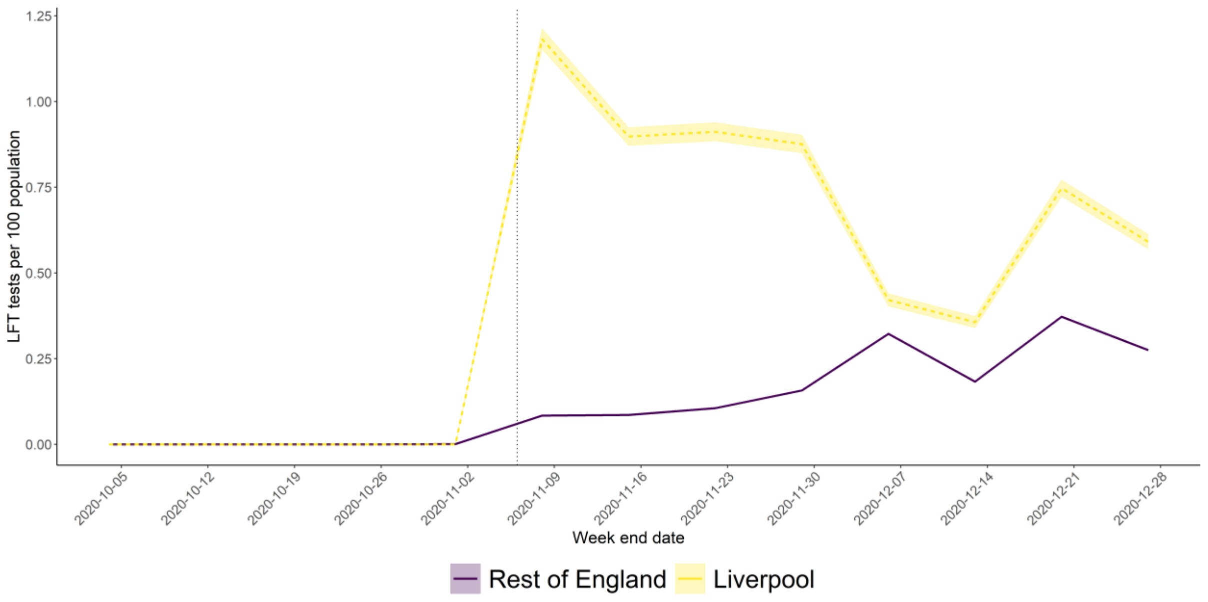

Figure SF2. Distribution of mean weekly LFT tests per 100 population across Local Authorities in England between 6th November 2020 and 2nd January 2021. Note: Dotted vertical line identifies the threshold of the mean LFT testing rate of 1 per 100 population per week that we used to exclude MSOAs from the control group to minimise the potential impact of similar asymptomatic pilot programmes elsewhere.

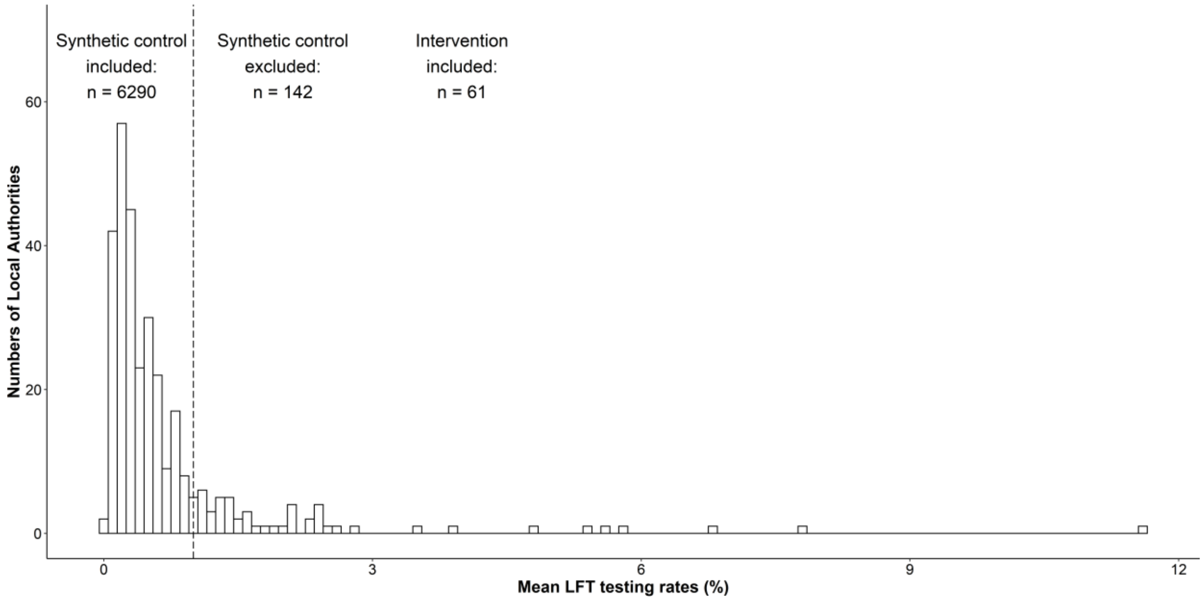

Part 2: Estimating Tier 3 restrictions on SARS-CoV-2 transmission compared to entering Tier 2 restrictions on 3 December 2020.

We analysed the impact of Tier 3 restrictions on SARS-CoV-2 transmission introduced in December 2020 in England, compared to Tier 2 restrictions. The main differences of Tier 2 and 3 were additional restrictions on meeting people outdoors and restrictions on the hospitality sector. To be more specific, in Tier 3 people were prohibited from meeting with people outside their household in private gardens whilst in Tier 2 people were allowed to meet with up to six people in private gardens; pubs and restaurants were closed in Tier 3 areas whilst in Tier 2 areas only those serving food remained open. We used data on COVID-19 restrictions that were compiled and made available by the Open Data Institute.[1]

As Liverpool introduced the less restrictive Tier 2 measures on 3rd December 2020, whilst new tier's restrictions typically took effect in many local authorities on the first forthcoming Monday (7th December 2020), we therefore specified 2 weeks after that point as the earliest plausible time at which the change in restrictions could affect hospital admissions (20th December 2020). We investigated the change in MSOA-level COVID-19 hospital admissions in the intervention group (Tier 3 areas) using synthetic control analysis, 12 weeks before and 10 weeks after that time point, compared to a synthetic control group derived from places that entered Tier 2 at the same time. The exact time frame ranged from 4 October 2020 to 21 February 2021. We identified 2,809 Tier 3 MSOAs as the intervention group, whilst the synthetic control group was constructed from the 3,481 Tier 2 MSOAs (excluding 61 MSOAs in the Local Authority of Liverpool).

Along with the local area characteristics outlined above in our analysis of COVID-SMART, we additionally accounted for differences in the prevalence of the new variant B.1.1.7 (Alpha), which became dominant during that time period, by including the proportion of positive tests with S-gene failure on PCR testing for each local authority in the period before the 20th December using data from Public Health England.[23] To further control for the confounding effect of community testing, we also excluded the 142 MSOAs (2.2% of all non-intervention MSOAs) from the control group if they were within authorities with a mean LFT testing rate of more than 1 per 100 population per

week between 6th November and 2nd January 2021. In the sensitivity testing in Part 5 of the Supplement, we provided the estimation of the Tier 3 effect upon hospitalisation, without excluding these 142 MSOAs.

The introduction of Tier 3 restrictions in December was associated with an average reduction of hospital admissions of 17% (95% CI 13% to 21%) in Tier 3 areas compared to Tier 2 areas. In the sensitivity testing in Part 5 of the Supplement, without excluding the 142 MSOAs with a mean LFT testing rate of more than 1 per 100 population per week during the pilot period of the community testing in Liverpool, the introduction of Tier 3 restrictions in December was associated with an average reduction of hospital admissions of 14% (95% CI 9% to 17%) in Tier 3 areas compared to Tier 2 areas.

As can be seen from Figure SF3 the effect of the Tier restrictions changes over time. In adjusting the MSOA hospital admissions in Tier 3 areas to account for this in our analysis of COVID-SMART, we applied the weekly effect sizes from the synthetic control analysis of Tiered restrictions.

Figure SF3. The effect of Tier 3 restrictions on COVID-19 hospital admissions relative to the Tier 2 synthetic control group.

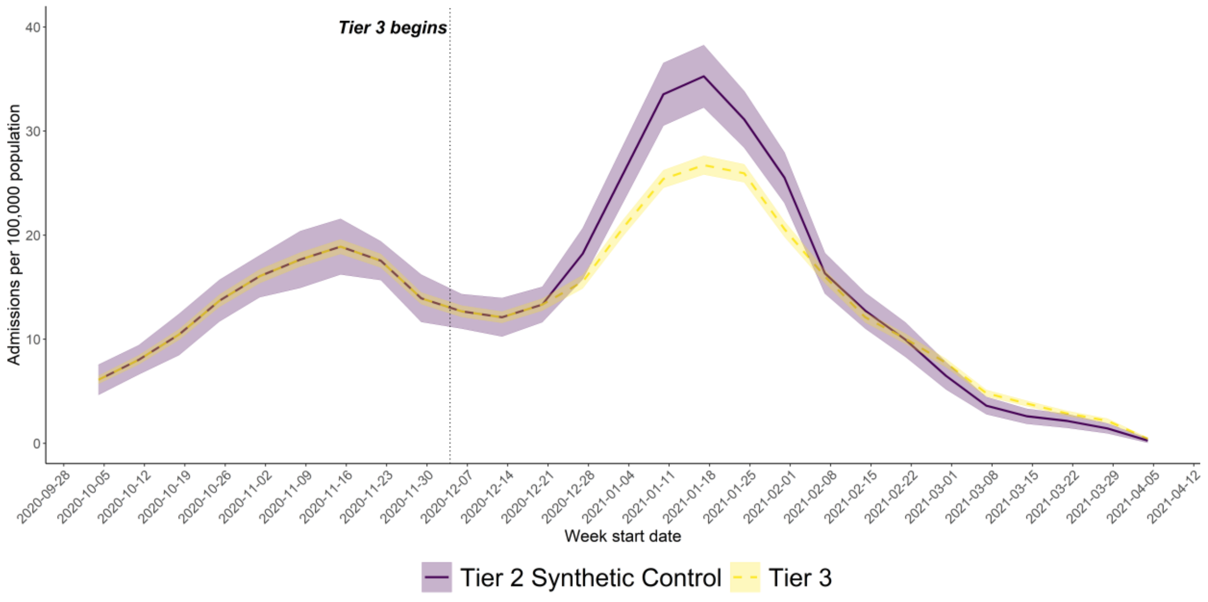

Part 3: Synthetic control area construct weighting.

Figure SF4 shows the geographical pattern of MSOAs used to construct the synthetic control group. The intervention group (61 MSOAs in Liverpool) is coloured in black, whilst the synthetic control group is constructed by applying the calibrated weights to various MSOAs across the country. Most of these MSOAs constituting the synthetic control group are in the North West region and along the North East coastlines.

Figure SF4. Weighting of areas used to construct the synthetic control group (Liverpool, coloured in black). The maps relate to a particular seed draw, with 54 MSOAs contributing: the map on the left shows the synthetic control MSOAs in their original locations and sizes; the map on the right shows the number of synthetic control MSOAs in each local authority to facilitate understanding. The major cities (along with their corresponding labels) are annotated with the same set of varying degree of cold colours (green/cyan/blue) respectively, whilst the weights are rendered in warm colours (yellow/red/purple).

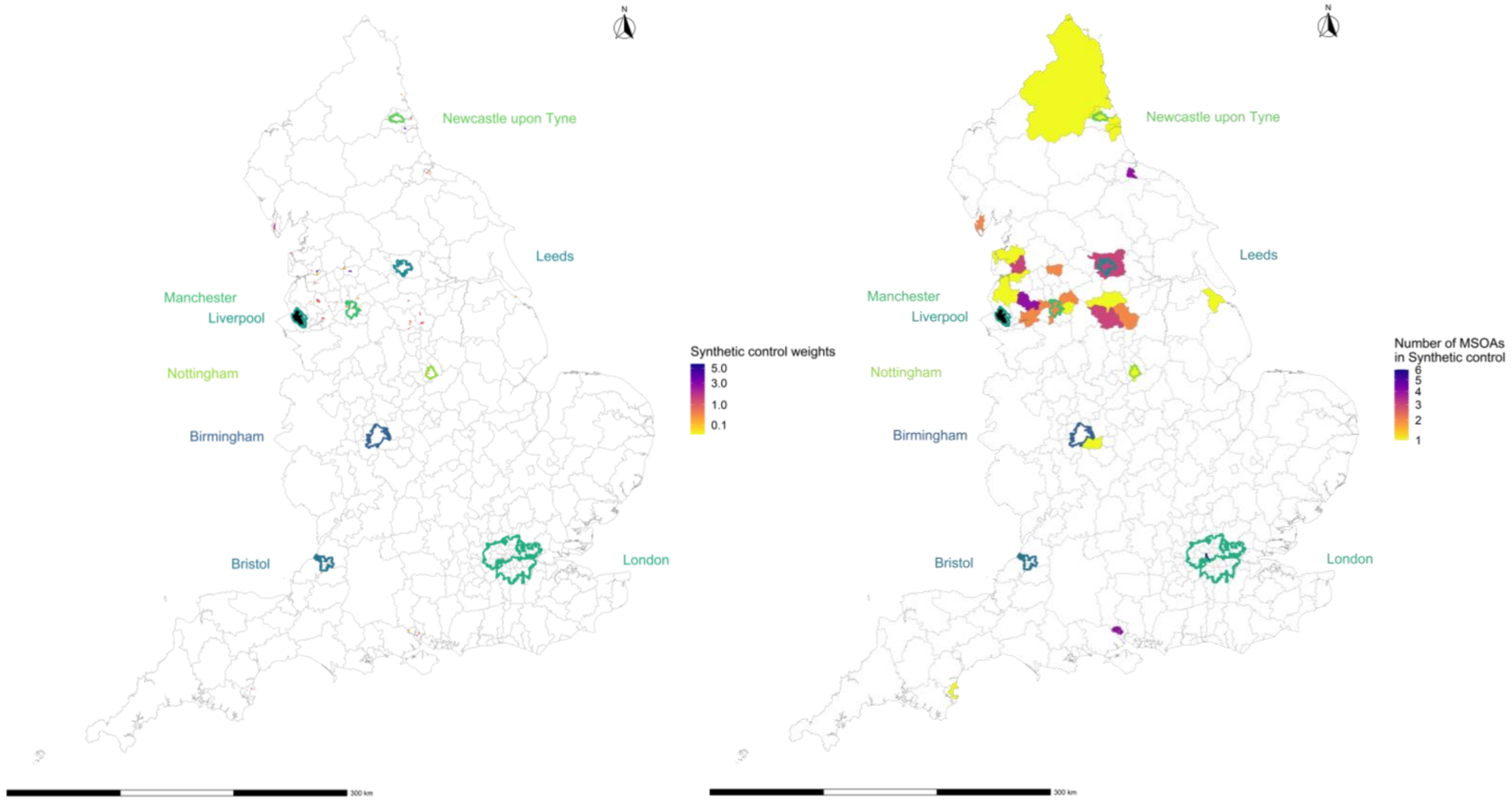

Part 4: Sensitivity test of adjusting the Tier 3 effect by the 95% upper and lower bound during the pilot period.

Table SF1. Estimated effect of community testing programme on COVID-19 hospital admissions from synthetic control analysis, adjusting the Tier 3 effect by the 95% upper and lower bound during the pilot period. This estimation is made under alternative assumptions related to the effect of the introduction of less stringent Tier 2 restriction in Liverpool in December 2021, excluding the 142 MSOAs with an LFT testing rate of more than 1 per 100 population per week during the pilot period. Model 3A and 3B are constructed by having adjusted with the upper and lower 95% CI bounds of the Tier 3 effect estimate respectively. Model 3A and 3B are linked to Model 3 in Table 2 of the main manuscript, which has been adjusted with the central estimate of the Tier 3 effect. Other than having been adjusted differently for the Tier 3 effect estimate, all three models (Model 3A, 3B and 3) cover the same study period and use the same modelling structure.

Model	Intervention period	Assumed reduction in COVID-19 hospital admissions from Tier 3 vs 2 restrictions	% Difference in COVID-19 hospital admissions between Liverpool and control	Lower 95% CL	Upper 95% CL	P-value
**3A**	6th November 2020 to 2nd January 2021	21% (upper 95% bound)	- 29%	- 39%	- 15%	<0.001
**3B**		13% (lower 95% bound)	- 22%	- 31%	- 7%	0.014

Part 5: Sensitivity analysis: not excluding MSOAs with high lateral flow testing.

Table SF2. Estimated effect of community testing on COVID-19 hospital admissions from synthetic control analysis, without excluding 142 MSOAs with LFT testing > 1 per 100 population per week from the control group. Model A, B and C are linked to Model 1, 2 and 3 respectively in Table 2, with each corresponding pair of models covering the same study period and using the same modelling structure. Model D and E are linked to Model 3A and 3B in Table SF1 respectively, which all are linked to Model 3 subsequently.

Model	Intervention period	Assumed reduction in COVID-19 hospital admissions from Tier 3 vs 2 restrictions	% Difference in COVID-19 hospital admissions between Liverpool and control	Lower 95% CL	Upper 95% CL	P- value
A	6th November to 3rd December 2020	Nil	- 45%	- 54%	- 34%	<0.001
B	6th November 2020 to 2nd January 2021	Nil	- 11%	- 22%	5%	0.182
C		14% (central estimate)	- 20%	- 29%	- 6%	<0.001
D		17% (upper 95% bound)	- 24%	- 33%	- 11%	<0.001
E		9% (lower 95% bound)	- 17%	- 26%	- 2%	0.040

Part 6: Detailed comparison of additional characteristics between Liverpool, the MSOAs in the rest of England used to construct the synthetic control group (i.e. excluding those within Liverpool City Region or with a high LFT testing rate), and the synthetic control.

Table SF3. Detailed comparison of characteristics between Liverpool and the MSOAs in the rest of England used to construct the synthetic control group (i.e. excluding those within Liverpool City Region or with a high LFT testing rate) prior to 19th November (preintervention period). Rows with doubly-lined borders are the seven matching local area characteristics used in constructing the synthetic control group.

	Liverpool			MSOAs in the rest of England used to construct the synthetic control			Synthetic control (weighting MSOAs in the rest of England using group-matching weights)		
Number of MSOAs	61			6290			61		
Total population	498042			52330147			498042		
	Mean	Min	Max	Mean	Min	Max	Mean	Min	Max
MSOA population	8165	5391	17515	8320	4854	26513	8165	0	57397
2019 IMD score	43	8	74	21	2	77	43	0	198
Population density[1]	55	9	119	36	0	287	55	0	283

**(people per hectare)**									
**% of population 70+[1]**	11	1	21	14	0	43	11	0	66
**% BAME[2]**	11	2	65	14	1	94	11	0	98
**% of population with at least 1 admission for a chronic disease[3]**	24	18	30	20	8	34	24	0	143
**Number of PCR tests per 100,000 population[4]**	3572	3572	3572	2552	1713	5359	3572	0	21821
**Average hospital admissions per 100,000 population per week for COVID-19[4]**	26	0	135	9	0	148	24	0	336
**Weekly COVID-19 cases per 100,000 population per week[4]**	464	70	2872	203	0	7240	344	0	3328

**2019 IMD score of education domain**	34	2	70	21	0	94	43	0	202
**% children (under 18)**	20	3	32	21	2	43	24	0	149
**% women**	50	38	55	51	36	56	51	0	285
**% urban population**	100	100	100	82	2	100	94	0	510
**Number of weeks in Tier 1**	0	0	0	0	0	0	0	0	0
**Number of weeks in Tier 2**	0	0	0	1	0	3	2	0	12
**Number of weeks in Tier 3**	3	3	3	0	0	2	1	0	11
**Number of weeks in Tier 4**	0	0	0	0	0	0	0	0	0

[1] calculated using 2019's mid-year population estimates from the Office for National Statistics

[2] data obtained from the 2011 Census

[3] based on HES data between 2014 and 2018

[4] data refer to the pre-intervention period from 5th October 2020 to 5th November 2020

Table SF4. Comparing the total number of COVID-19 hospital admissions (100,000 persons) in the control group and Liverpool for each of the seven weeks prior to 19th November (preintervention period).

Weeks before intervention on 19th November 2020	Liverpool	MSOAs in the rest of England used to construct the synthetic control	Synthetic control (weighting MSOAs in the rest of England using group-matching weights)
7	128	2004	128
6	172	2572	172
5	153	3434	153
4	136	4549	136
3	126	5474	126
2	93	5956	93
1	63	6644	63

Part 7: Sensitivity test of local authority level synthetic control analysis.

Our approach using the synthetic control method for micro data developed by Robbins et al,[3] aggregates a large number of small areas to provide a synthetic control group similar to Liverpool. As we note our synthetic control group was made up of a non-adjacent set of neighbourhoods, that in aggregate had followed similar trends in hospitalisation to the contiguous neighbourhoods of Liverpool. However, this could introduce bias as a combination of those smaller areas may not mimic rates in a contiguous urban area. We therefore conducted a sensitivity analysis at the local authority level, matching Liverpool to 10 other local authority areas that have similar characteristics and previous trends in hospital admissions.

As for this analysis we have just one intervention unit, this presents estimation complications in deriving a relevant comparison group and for calculating standard errors. We therefore use the synthetic control approach developed by Brodersen et al,[4] which has previously been used to evaluate local authority level policies with single intervention units.[5] The synthetic controls are calculated using Bayesian structural timeseries based on weighted combinations of the control areas. The approach uses Bayesian model averaging of the time series in all control areas to create a synthetic time series which is similar to the measured time series in Liverpool prior to the intervention and a post-intervention synthetic time series predicting what would have happened in the absence of COVID-SMART (i.e., the counterfactual). Bayesian priors are placed on the regression coefficients of all control areas included in the preintervention model. The semiparametric Bayesian posterior distribution for the effect of COVID-SMART is obtained as the difference between the measured outcomes in Liverpool and the counterfactual time series post intervention. The standard deviation of the regression coefficients was set to 10% of the preintervention outcomes. The results are presented as point estimates and Bayesian 95% credible intervals (CIs).

The control local authorities for this analysis were selected using Mahalanobis nearest neighbour matching,[6,7] matching on the same variables included in our main analysis aggregated to the local authority level and applying the same exclusion criteria. This provided 10 local authorities that were included in the comparison group:

Stockton-on-Tees, County Durham, Burnley, Preston, West Lancashire, Wyre, Bury, Stockport, Liverpool, Newcastle upon Tyne and Sunderland. We used the same intervention period from 6th November to 3rd December 2020 in this analysis as in our main analysis.

The results in Table SF5 indicate an effect size that is consistent with our main analysis using small area data, with the intervention during the initial period being associated with a 68% reduction in hospital admissions (95% credible intervals 116% to 18% reduction, p=0.009). Figure SF5 shows the trend in Liverpool and the estimated counterfactual trend and Figure SF6 shows the cumulative difference between the two.

Table SF5. Estimated effects of COVID-SMART community testing on COVID-19 hospital admissions from local authority level synthetic control analysis.

**Model**	**Intervention period**	**Assumed reduction in COVID-19 hospital admissions from Tier 3 vs 2 restrictions**	**% Difference in COVID-19 hospital admissions between Liverpool and control**	**Lower 95% credible interval**	**Upper 95% credible interval**	**P- value** (Posterior tail-area probability)
**LA**	6th November to 3rd December 2020	Nil	-68%	-116%	-18%	0.009

Figure SF5. Trend in weekly COVID-19 hospital admission rates in Liverpool City (yellow dashed line) compared to a synthetic control group (the counterfactual; purple solid line) constructed from 10 comparable local authorities without community testing. The dotted vertical line represents the start of the (lagged) intervention period on 19th November 2020.

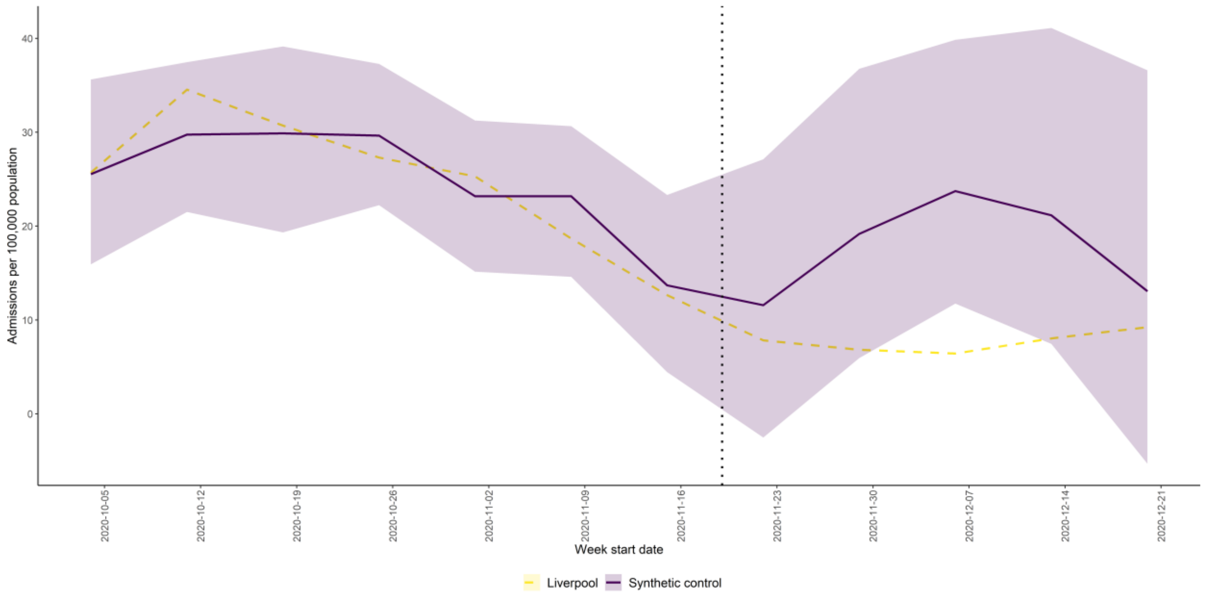

Figure SF6. The cumulative difference in hospital admissions between Liverpool (yellow dashed line) and the counterfactual (purple solid line) constructed from 10 comparable local authorities without community testing during the intervention period. The dotted vertical line represents the start of the (lagged) intervention period on 19th November 2020.

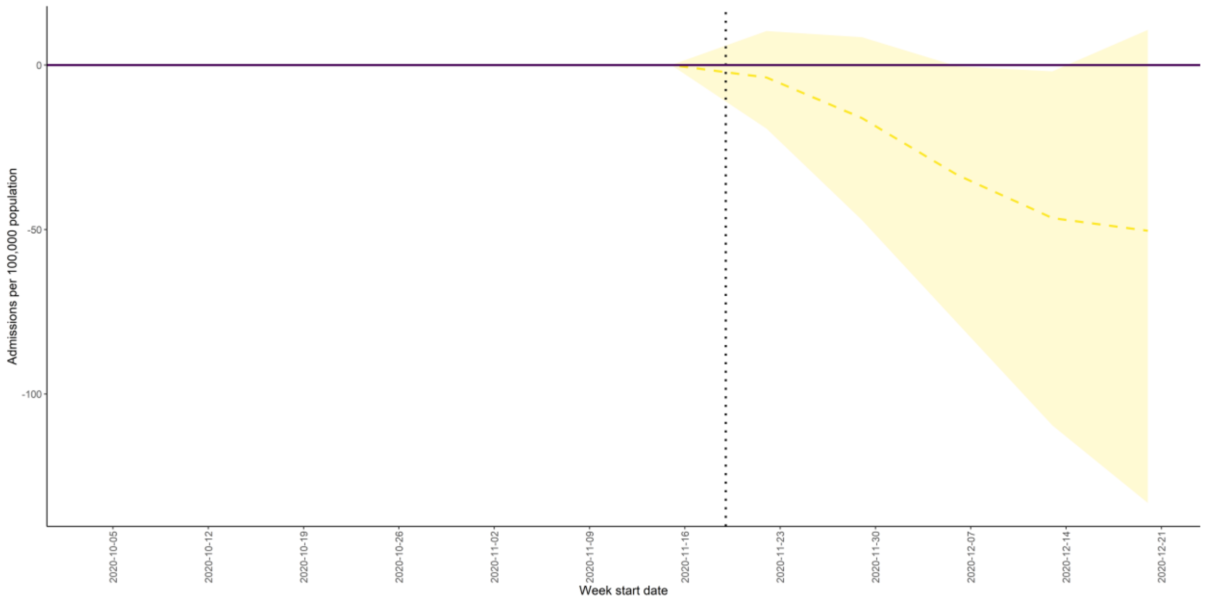

Part 8: Sensitivity test of adding the proportion of women in the matching.

Table SF6. Estimated effects of COVID-SMART community testing on COVID-19 hospital admissions from synthetic control analysis after adding the proportion of women in the matching, under alternative assumptions over the effects of a lower level of restrictions in Liverpool City in December 2020. Model Sex1, Sex2 and Sex3 correspond to Model 1, 2 and 3 respectively in Table 2 of the main manuscript.

Model	Intervention period	Assumed reduction in COVID-19 hospital admissions from Tier 3 vs 2 restrictions	% Difference in COVID-19 hospital admissions between Liverpool and control	Lower 95% CL	Upper 95% CL	P- value
**Sex1**	6th November to 3rd December 2020	Nil	-43%	-57%	-28%	<0.001
**Sex2**	6th November 2020 to 2nd January 2021	Nil	-14%	-27%	1%	0.093
**Sex3**		17% (central estimate)	-24%	-34%	-9%	<0.001

Part 9: Sensitivity test of using the score of Education Skills and Training Deprivation rather than the composite score of IMD in the matching.

Table SF7. Estimated effects of COVID-SMART community testing on COVID-19 hospital admissions from synthetic control analysis using the score of Education Skills and Training Deprivation rather than the composite score of IMD in the matching, under alternative assumptions over the effects of a lower level of restrictions in Liverpool City in December 2020. Model Edu1, Edu2 and Edu3 correspond to Model 1, 2 and 3 respectively in Table 2 of the main manuscript.

Model	Intervention period	Assumed reduction in COVID-19 hospital admissions from Tier 3 vs 2 restrictions	% Difference in COVID-19 hospital admissions between Liverpool and control	Lower 95% CL	Upper 95% CL	P- value
**Edu1**	6th November to 3rd December 2020	Nil	-46%	-57%	-34%	<0.001
**Edu2**	6th November 2020 to 2nd January 2021	Nil	-17%	-28%	-2%	0.043
**Edu3**		17% (central estimate)	-26%	-36%	-12%	<0.001

References

1 Tennison J. Local coronavirus restrictions in England. Google Docs. https://docs.google.com/spreadsheets/d/1HBVmvsQXrkQgySW_OiTQdrS8WGCXqgWnmZ43PPi0XgY/edit?usp=embed_facebook (accessed 28 Jan 2021).

2 Kirby T. New variant of SARS-CoV-2 in UK causes surge of COVID-19. *Lancet Respir Med* 2021;**0**. doi:10/ghwffp

3 Robbins MW, Davenport S. microsynth: Synthetic Control Methods for Disaggregated and Micro-Level Data in R. *J Stat Softw Vol 1 Issue 2 2021* Published Online First: 14 January 2021.https://www.jstatsoft.org/v097/i02

4 Brodersen KH, Gallusser F, Koehler J, *et al.* Inferring causal impact using Bayesian structural time-series models. *Ann Appl Stat* 2015;**9**. doi:10/gc5p78

5 Vocht F de, Tilling K, Pliakas T, *et al.* The intervention effect of local alcohol licensing policies on hospital admission and crime: a natural experiment using a novel Bayesian synthetictime-series method. *J Epidemiol Community Health* 2017;**71**:912–8. doi:10/gm8g3q

6 Thoemmes FJ, Kim ES. A Systematic Review of Propensity Score Methods in the Social Sciences. *Multivar Behav Res* 2011;**46**:90–118. doi:10.1080/00273171.2011.540475

7 Zakrison TL, Austin PC, McCredie VA. A systematic review of propensity score methods in the acute care surgery literature: avoiding the pitfalls and proposing a set of reporting guidelines. *Eur J Trauma Emerg Surg* 2018;**44**:385–95. doi:10.1007/s00068-017-0786-6
